# Different categories of biodiversity explain productivity variation after fertilization in a Tibetan alpine meadow community

**DOI:** 10.1002/ece3.2723

**Published:** 2017-04-06

**Authors:** Xiaolong Zhou, Zhi Guo, Pengfei Zhang, Honglin Li, Chengjin Chu, Xilai Li, Guozhen Du

**Affiliations:** ^1^ State Key Laboratory of Grassland and Agro‐ecosystemsSchool of Life SciencesLanzhou UniversityLanzhouGansuChina; ^2^ Institute of Arid Ecology and EnvironmentXinjiang UniversityUrumqiXinjiangChina; ^3^ SYSU‐Alberta Joint Lab for Biodiversity ConservationState Key Laboratory of Biocontrol and School of Life SciencesSun Yat‐sen UniversityGuangzhouChina; ^4^ College of Agriculture and Animal HusbandryQinghai UniversityXiningChina

**Keywords:** alpine meadow, functional diversity, nitrogen and phosphorus fertilization, phylogenetic diversity, productivity, species diversity

## Abstract

The relationship between productivity and biodiversity has long been an important issue in ecological research. However, in recent decades, most ecologists have primarily focused on species diversity while paying little attention to functional diversity and phylogenetic diversity (PD), especially in alpine meadow communities following fertilization. In this study, a fertilization experiment involving the addition of nitrogen, phosphorus, and a mixture of both was implemented in an alpine meadow on the Tibetan Plateau. Species diversity, functional diversity, and PD were measured, and the responses of these parameters to the variation in productivity were analyzed. We found that the productivity of alpine plant communities was colimited by N and P, with N being the principal and P being the secondary limiting nutrient. Our results supported the prediction of both the mass ratio hypothesis and niche complementarity hypothesis in fertilized communities, but these hypotheses were not mutually exclusive. The combination of different aspects of biodiversity not only provides a crucial tool to explain the variation in productivity and to understand the underlying mechanisms but also plays an important role in predicting the variation in productivity of alpine meadow communities, which are sensitive to nutrient enrichment in the context of global change.

## 1 Introduction

For more than 30 years, the pattern of the relationship between productivity and biodiversity has been a widely debated topic in ecology (Adler et al., 2011; Isbell et al., 2013; Kessler, Salazar, Homeier, & Kluge, 2014). In natural communities, the pattern has traditionally been considered to be hump‐shaped (Grace, 1999; Huston, 1979), but positive (Gaitán et al., 2014; Tilman, Wedin, & Knops, 1996; Tilman et al., 2001) and nonsignificant relationships (Adler et al., 2011; Grace, Adler, Harpole, Borer, & Seabloom, 2014) have also been reported. A negative productivity –biodiversity relationship has been found in many fertilization experiments (Crawley et al., 2005; Dickson & Gross, 2013; Gerstner, Dormann, Stein, Manceur, & Seppelt, 2014), and two nonexclusive hypotheses have been proposed to explain this phenomenon: the mass ratio hypothesis, which states that productivity is strongly influenced by the character of the dominant species in a community (Grime, 1998; Hooper & Vitousek, 1997; Mokany, Ash, & Roxburgh, 2008; Winfree, Fox, Williams, Reilly, & Cariveau, 2015), and the niche complementarity hypothesis, which assumes that combinations of species are complementary in the types of resources they use and thus increase community productivity (Tilman, Knops, et al., 1997; Wilsey & Potvin, 2000). Grassland productivity is key to the provision of food for domestic herbivores and is known to depend on biodiversity, the functional characteristics of a community, and nutrient limitations (Borer et al., 2014; Crawley et al., 2005; Goldberg & Miller, 1990; Onipchenko et al., 2012; Ren et al., 2010). Therefore, determining how productivity varies and how it is affected by biodiversity, community functional characteristics and nutrient availability in this area are important to both theory and practice. Although the productivity–biodiversity relationship has been well studied in both natural and artificially fertilized communities over the past several decades, numerous questions remain (Hooper et al., 2005; McClain et al., 2015). One of the most important reasons for this uncertainty is that biodiversity is a multifaceted concept that encompasses different categories such as species, functional diversity, and phylogenetic diversity (PD; Cadotte, Cavender‐Bares, Tilman, & Oakley, 2009; Díaz, Fargione, Chapin, & Tilman, 2006; Willig, 2011), but in previous studies, researchers have primarily focused on species diversity while paying little attention to functional diversity and PD (Crawley et al., 2005; Gough, Osenberg, Gross, & Collins, 2000; Hector et al., 1999; Jucker et al., 2015). In fact, the roles of different species within a community are not equal, as is assumed by species diversity theory; in contrast, species differ in their functional traits and phylogenetic histories, which have a greater impact on productivity (Loreau, Naeem, & Inchausti, 2002; Mouchet, Villéger, Mason, & Mouillot, 2010; Paquette, Joly, & Messier, 2015; Tilman, Knops, et al., 1997). Moreover, following fertilization, species diversity has generally been found to decrease, but no consistent tendency has been observed in functional diversity (Bello et al., 2013; Li et al., 2015; Niu et al., 2013). Therefore, species diversity is not an appropriate surrogate for functional diversity in many cases (De Bello et al., 2009; Li et al., 2015), which may explain the finite variation in productivity in different communities, especially after fertilization (Grace et al., 2007; Klaus et al., 2013).

Over the past several decades, many studies have demonstrated that functional diversity and PD play an important role in explaining the variation in the productivity of plant communities. Tilman, Knops, et al. (1997), Tilman, Lehman, and Thomson (1997) reported that both species diversity and functional diversity have significant effects on ecosystem processes, but functional diversity is the principal factor explaining plant productivity. Cadotte et al. (2009) found the PD best explains community productivity patterns in comparison with species diversity and functional diversity, while Niu et al. (2013) revealed a negative relationship between community biomass and species diversity but a positive correlation between community biomass and functional diversity following fertilization of an alpine meadow. A study in Mediterranean rangelands showed that the combination of functional diversity and abiotic variables could predict 80% of the biomass produced, but those controls depend on the season (Chollet et al., 2014). In a forest ecosystem, Lohbeck, Poorter, Martínez‐Ramos, and Bongers (2014a) found that functional diversity and the community‐weighted mean (CWM) of certain traits could provide additional power to explain biomass production and potential decomposition. Liu et al. (2014) found that PD and plant height represent the most parsimonious combination to explain productivity in an alpine meadow of the Tibetan Plateau, and Li et al. (2015) concluded that the CWM of plant height is positively correlated with the productivity of a fertilized community. In conclusion, to explore the effects of biodiversity on productivity, ecologists should use appropriate biodiversity indices, which might involve a combination of species diversity, functional diversity, and PD (Reiss, Bridle, Montoya, & Woodward, 2009).

To date, many studies have been conducted to answer these questions, and they have yielded mixed results (Li et al., 2015; Liu et al., 2014; Niu, Luo, Choler, & Du, 2008; Zhou et al., 2015). However, most of the previous studies focused on species diversity, but few investigated a combination of species diversity, functional diversity, and PD to explain and predict the variation in productivity. In addition, some studies found that nitrogen is the limiting nutrient in soil (Li, Wen, Hu, & Du, 2011; Ren et al., 2010), but others stated that phosphorus limits productivity on the Tibetan Plateau (Niu, Messier, He, & Lechowicz, 2015). We systematically tested 16 diversity indices associated with species diversity, functional diversity, and PD to explain and predict the variation in community productivity following the addition of N, P, and N + P. To our knowledge, this is the first study to systematically study the relationships between productivity and three different aspects of biodiversity (SD, PD, and FD) through an N and P fertilization experiment in this region. Specifically, we asked the following questions: (1) Which nutrient, nitrogen or phosphorus, limits productivity in alpine meadows? (2) Among the diversity measures considered, which is the best and how can they explain the variation in productivity after fertilization in combination? (3) What is the mechanism underlying these patterns?

## 2 Materials and Methods

### 2.1 Study site

**Figure 1:**
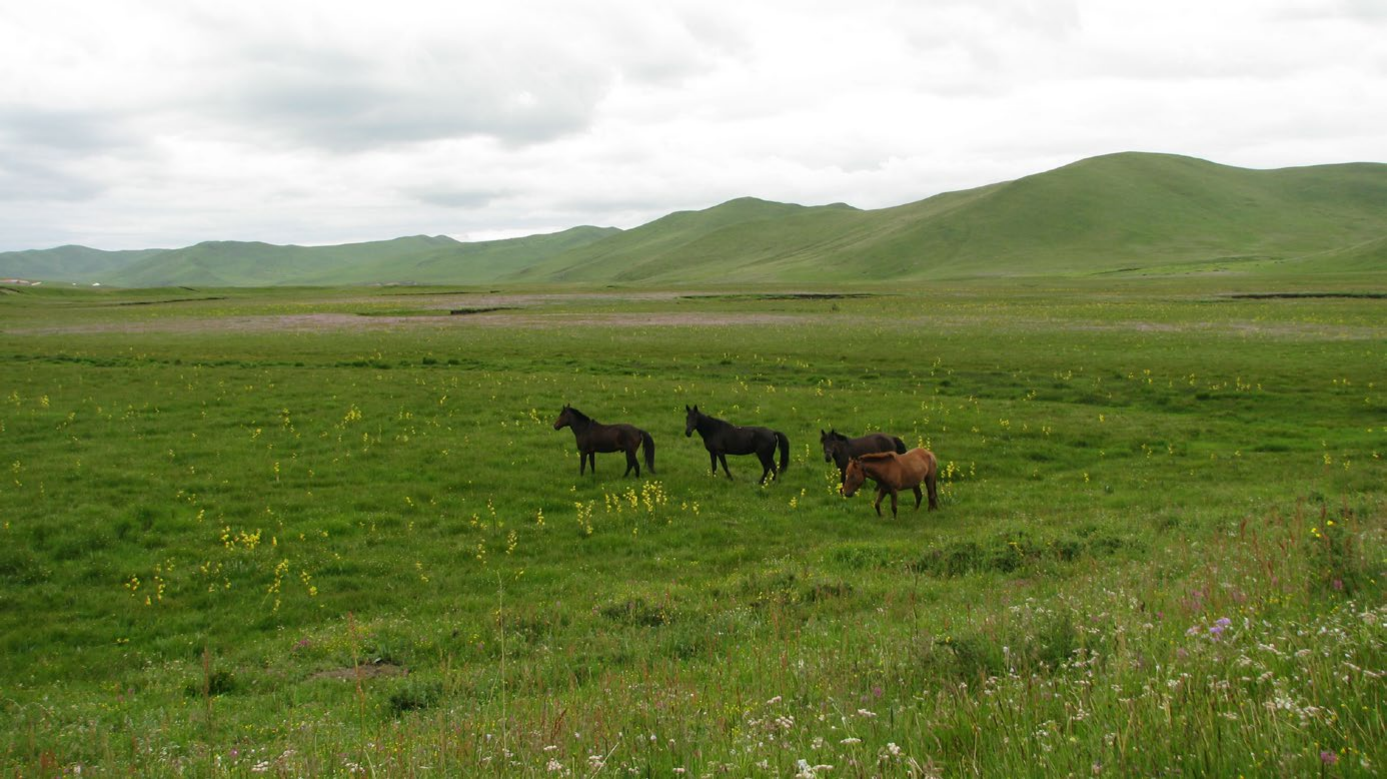
The alpine meadow on the Tibetan Plateau, which is one of the most sensitive and vulnerable terrestrial ecosystems to anthropogenic nutrient enrichment

The Tibetan Plateau, the largest geomorphologic unit on the Eurasian continent, has an average altitude of more than 4,000 m a.s.l. and covers approximately 2.5 million km^2^, of which 35% are alpine meadows. The plateau, which is one of the most sensitive and vulnerable terrestrial ecosystems to climate change, is also one of the largest rangeland areas in the world (Klein, Harte, & Zhao, 2007; Figure 1). The study was carried out at the Alpine Meadow and Wetland Ecosystems Research Station of Lanzhou University (Maqu branch) in the eastern Tibetan Plateau (35°580′N, 101°530′E, altitude 3,500 m a.s.l.), Gansu, China. The mean annual temperature is 1.2°C, ranging from −10°C in January to 11.7°C in July, and the mean annual precipitation for the 1975–2010 period was 620 mm, which mainly fell during the short, cool summer. The area has 2,580 hr of sunshine and more than 270 days of frost per year (Luo, Qin, & Du, 2006). The vegetation is that of a typical alpine meadow and is dominated by *Kobresia graminifolia* (Cyperaceae), *Elymus nutans* (Poaceae), *Anemone rivularis* (Ranunculaceae), *Poa poophagorum* (Poaceae), *Festuca ovina* (Poaceae), and *Carex kansuensis* (Cyperaceae), and the average aboveground dry biomass is 360–560 g/m^2^. The dominant animals in the area include livestock (e.g., yaks, Tibetan sheep, and horses), marmots (*Marmota himalayana*), zokor (*Myospalax* spp.), and various ant species.

### 2.2 Experimental design

We conducted a fertilization experiment in an alpine meadow that was arranged in three blocks: nitrogen (N) fertilization, phosphorus (P) fertilization, and N + P mixed fertilization. There were three different fertilization (treatment) levels in each block: 5, 10, and 15 g/m^2^ in the N fertilization block; 2, 4, and 8 g/m^2^ in the P fertilization block; and 10 g/m^2^ N + 2 g/m^2^ P, 10 g/m^2^ N + 4 g/m^2^ P, and 10 g/m^2^ N + 8 g/m^2^ P in the N + P mixed fertilization block. In total, there were nine fertilization treatments and one control treatment (without any fertilization), and each treatment was replicated six times.

The fertilization experiment was established in April 2011 in an enclosed area of flat alpine meadow, where grazing was only allowed during the nonproductive winter. In late May 2011, 60 20 × 10 m plots, each separated by 1 m, were established in a 230 × 100 m area of homogeneous meadow. Then, each plot was divided into two 10 × 10 m subplots; one subplot was used to measure species abundance, and the other subplot was used to measure functional traits. In subsequent years, fertilizer was applied annually at the end of May on drizzly days to avoid the need for watering.

### 2.3 Species abundance measurements

At the middle of August 2015 (after 5 years of fertilization), a 0.5 × 0.5 m quadrat was harvested from each treatment replicate (60 quadrats in total), and the number of individuals of each species was counted. For clonal plants, the term individual refers to a ramet (Cheplick, 1989), which are equivalent to tillers in graminoids and rosettes or rooting branches in forbs. The green, aboveground parts (stems and leaves) were then clipped and sorted by species and brought to the laboratory. The green parts were dried at 60°C for 48 hr and weighed (0.01 g) to estimate biomass productivity.

### 2.4 Functional trait measurements

Following the leaf–height–seed plant ecology strategy scheme (Westoby, 1998), we chose six functional traits: height, specific leaf area (SLA), leaf dry mass content (LDMC), leaf N content, leaf P content, and seed mass, all of which can be easily measured and have important ecological meaning in our study. In 2015, these six functional traits were measured in 21 common species (Appendix S1) following the flowering phase. These 21 species accounted for 70%–90% of the aboveground biomass, and for each species, we randomly sampled nine fully developed and undamaged leaves. The fresh leaves were weighed before being scanned to measure leaf area using ImageJ software (Schneider, Rasband, & Eliceiri, 2012); the leaves were then dried at 70°C for 48 hr and weighed using a Sartorius balance with an accuracy of 10^*−*4^ g. We calculated SLA as the ratio of leaf area to dry leaf mass and LDMC as the ratio of dry leaf mass to fresh leaf mass. We also randomly selected 30 flowering individuals of each species to measure the species‐saturated height in each treatment; we then clipped the plants, dried them at 70*°*C for 48 hr, divided them into leaves, stems, and flowers, and weighed them using the Sartorius balance. Next, the leaves were used to measure the nitrogen and phosphorus contents. In the laboratory, the leaves were first ground and oven dried at 60°C for 48 hr to a constant weight, and the N and P concentrations in the leaves were analyzed using a continuous flow‐injection analyzer (SKALAR, Breda, the Netherlands). The N and P contents were calculated per unit leaf dry mass. Additionally, we collected approximately 400 mature seeds from 20 to 30 fruiting individuals of each species in unfertilized control plots. Three replicates of 100 dried seeds from each species were weighed to measure seed mass.

### 2.5 Analysis of soil properties

Five main soil physical and chemical characteristics were measured in the laboratory and the field. Soil pH was measured with a glass electrode in a 1:2.5 soil:water solution, and the available P was determined by the molybdenum blue method. The available N that included two components, NH_4_^+^ and NO_3_^−^, was extracted with 2 mol/L KCl and measured using a continuous flow‐injection analyzer (SKALAR). During the growing season, soil temperature (°C) and soil moisture (m^3^/m^3^) were continuously recorded in the plots with the different fertilization treatment using data loggers (Em50 Decagon Devices Inc., Washington, DC, USA), and we measured the mean soil temperature and soil moisture in August.

### 2.6 Data analysis

Firstly, we calculated the species diversity, including richness, the Shannon index, the Simpson index, and evenness based on species biomass. Secondly, the most relevant functional diversity components were calculated as the CWM trait values ($\text{CWM}=\sum_{i=1}^{S}P_{i}\times {\text{trait}}_{i}$, here *P*_*i*_ is the relative abundance based on biomass; trait_*i*_ is the mean trait value of species *i*;* S* is community species richness; Garnier et al., 2004); and the different dimensions of functional diversity were summarized by three families of metrics: functional richness (FRic), functional evenness (FEve), and functional divergence (FDiv; Bello et al., 2013; Villéger, Mason, & Mouillot, 2008). We calculated different aspects of functional diversity using a series of indexes including FRic, FEve, FDiv, and six CWM traits (CWM height, CWM LDMC, CWM SLA, CWM seed size, CWM leaf N, and CWM leaf P). Thirdly, based on the published phylogenetic supertree of angiosperm families and APG III, we built a phylogenetic tree for species of interest with Phylomatic and Phylocom (Webb, Ackerly, & Kembel, 2008; Webb & Donoghue, 2005) and measured three indexes: PD, mean phylogenetic distance (MPD), and mean nearest taxon phylogenetic distance (MNTD). To calculate species diversity, we used the vegan package (Oksanen et al., 2015) developed for the statistical software R (R Development Core Team, 2013), and to calculate PD and functional diversity, we used the ape package (Paradis, Claude, & Strimmer, 2004), the picante package (Kembel et al., 2010), and the FD package (Laliberté & Legendre, 2010).

Before analyzing the effect of fertilization on biodiversity and the relationships between productivity and biodiversity, all variables were tested for the assumption of normality and homogeneity of variance using a Shapiro–Wilk test and a Levene test, respectively. For the data that were not normally distributed, a log10 (1 + *x*) transformation was used. Firstly, one‐way ANOVA was performed to determine the effects of different levels of fertilization on community productivity, species diversity, functional diversity, and PD, and post hoc comparisons among the different treatments were made using a Tukey's honest significant difference test. Secondly, we used a simple regression to estimate the relationships between community productivity and 16 biodiversity indexes, and a series of generalized linear models were employed to further evaluate the relationships between community productivity and a combination of biodiversity indexes. We only included significant biodiversity indexes in our analysis and selected the indexes based on the variance inflation factor (VIF). We gradually deleted the variables with largest VIF values to ensure that all variables were with low collinearity (VIF < 10). Model selection was based on the Akaike information criteria (AIC); given a set of competing models, we selected the one with the minimum AIC values. Thirdly, to elucidate the influence of fertilization on community composition, the total species community matrix were ordinated by principal components analysis (PCA) with a Euclidean measure. To explore the correlations between plant community and the corresponding environmental variables, soil variables were fitted as vectors in PCA plots. Finally, to determine the effects of different levels of fertilization and biodiversity on productivity, we constructed and tested a structural equation model (SEM), in which different categories of biodiversity (SD, FD, and PD) were represented by the scores of the first principle component of the series of indices (Appendix S2), respectively (Liu et al., 2012; Lohbeck et al., 2014a). We proposed a hypothetical model (Appendix S3) based on a priori knowledge and tested how well the model fits the data using the maximum likelihood χ^2^ goodness‐of‐fit test, the comparative fit index (CFI), and the Tucker–Lewis index (TLI). All statistical analyses were performed in R; the correlation analysis was conducted in the psych package (Revelle, 2015), and the generalized linear model selection was performed in the MASS package (Venables & Ripley, 2002). The PCAs were carried out using the vegan package, and SEM was conducted in the lavaan package (Rosseel, 2012).

## 3 Results

### 3.1 The shift in community composition and soil properties after fertilization

**Figure 2:**
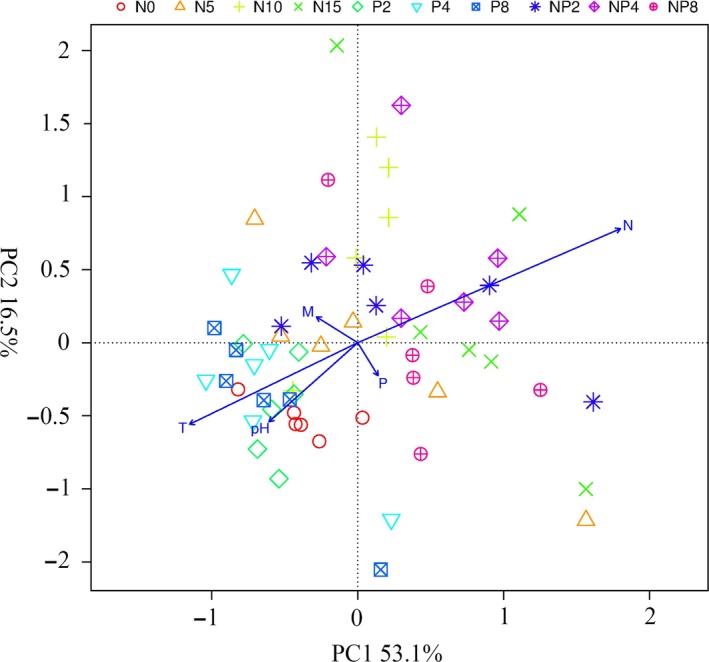
The principal components analysis to detect the effect of fertilization on community composition. The blue arrows and letters represent environment variables (N: soil available nitrogen, P: soil available phosphorus, T: soil temperature in August, M: soil moisture (v %) in August, pH: soil pH)

After 5 years of fertilization, we found that N addition increased the abundance of grasses but decreased forb abundance, thus severely changing species order and community composition (Appendix S1). However, we did not find significant effects of P fertilization on those community characters (Figure 2), and the results of community ordination revealed a strong difference in composition between the N fertilization and control communities (Figure 2). There was a shift in the plant communities from a mixture of forbs (*Anemone obtusiloba*,* A. rivularis, Oxytropis kansuensis, Thermopsis lanceolata*), grasses (*Poa crymophila*,* Poa pratensis*,* E. nutans*), and sedges (*K. graminifolia*) without N addition to a community dominated by two tall grass species (*P. pratensis*,* E. nutans*) with N addition. Community composition was not significantly different among the low N fertilization, middle N fertilization, high N fertilization, and N + P mixed fertilization treatments, so community composition remained similar to the control even after 5 years of P fertilization.

Fertilization affected the relationships among different soil properties. There was a strongly negative relationship between the available N and soil temperature (Figure 2), and a similar relationship was also apparent between the available N and soil pH (Figure 2).

### 3.2 The effect of fertilization on community productivity and biodiversity

**Figure 3:**
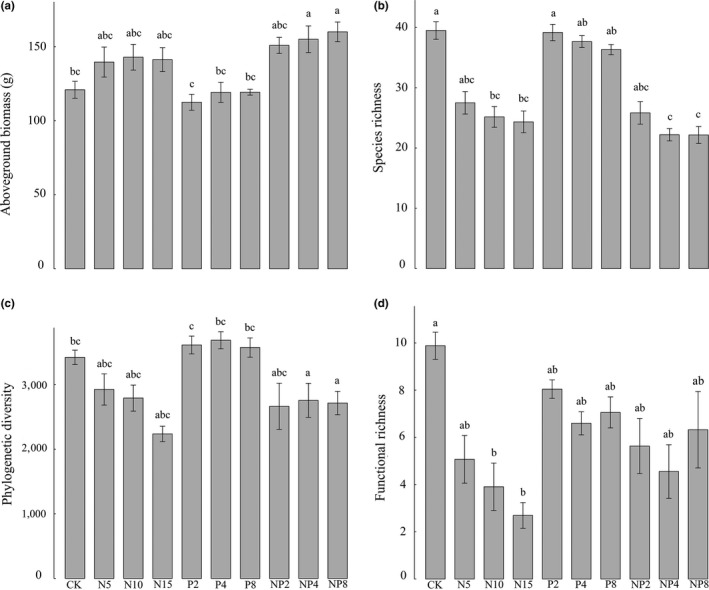
The effects of different fertilization on (a) aboveground biomass, (b) species richness, (c) Faith's phylogenetic diversity, and (d) functional richness. Different lowercase letters indicate significant differences (Tukey's test, *p* < .05) for the effect of fertilization in different types and doses

Neither N nor P fertilization alone significantly affected community productivity in our study, but the N + P mixed fertilization treatment significantly increased community productivity (Figure 3a). These results suggest that there might be a strong N fertilization and P fertilization interaction effect. Compared with the control, N fertilization significantly decreased species richness, PD, and FRic (Figure 3b–d), while SD, PD, and FD did not change under P fertilization. The change in SD, PD, and FD with N + P mixed fertilization treatments was analogous to that under the middle level of N fertilization (Figure 3b–d).

**Figure 4:**
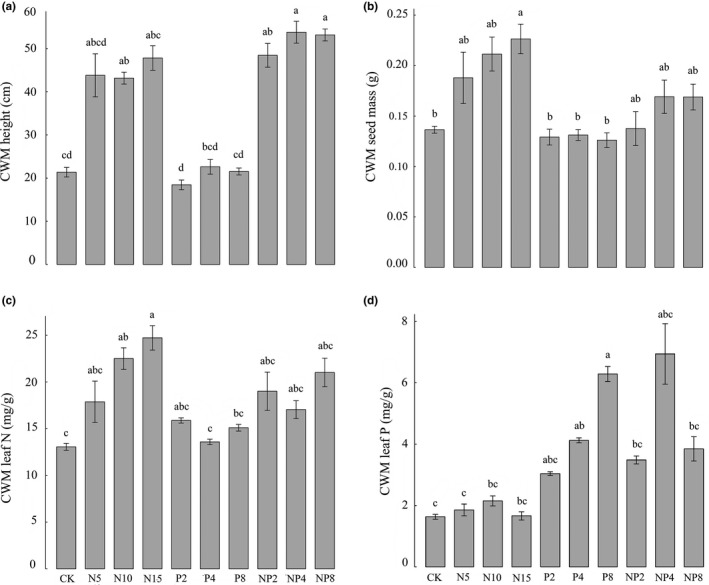
The effects of different fertilization on (a) CWM height, (b) CWM seed size, (c) CWM leaf nitrogen content, and (d) CWM leaf phosphorus content. Different lowercase letters indicate significant differences (Tukey's test, *p* < .05) for the effect of fertilization in different types and doses

CWM height, CWM seed size, and CWM leaf N significantly increased both with N fertilization and N + P mixed fertilization, while CWM leaf P only significantly increased in the P fertilization treatments (Figure 4).

### 3.3 The relationships between community productivity and SD, PD, and FD

Community productivity was negatively correlated with species richness, the Shannon index, and the Simpson index after 5 years of fertilization (Table 1), and the relationships between community productivity and PD, MPD, and MNTD were also negative (Table 1). One important explanation for this result was that PD was positively related to SD, which was supported by the correlation results (Appendix S4). The relationship between community productivity and FD was complicated. There were negative relationships between community productivity and FRic and FEve but a positive relationship between community productivity and FDiv (Table 1). All of the CWM traits (CWM height, CWM LDMC, CWM SLA, CWM seed size, CWM leaf N, and CWM leaf P) were positively correlated with community productivity, but the relationship between productivity and CWM SLA and CWM leaf P was not significant (Table 1). Finally, we constructed a series of generalized linear models (Appendix S5) and proposed the following model (minimum AIC) to best explain productivity using a combination of different biodiversity indices: productivity = 161.41 − 1.98 richness + 2.87 FDiv + 0.45 CWM height (*R* ^2^ = .568, *p* < .001).

**Table 1:** The simple regression results between community aboveground biomass and different biodiversity indices

	Indices	Intercept	Slope	*R* ^2^	*p*
Species diversity	Richness	196.81	−2.03	.4652	**<.001**
	Shannon index	207.97	−35.66	.3082	**<.001**
	Simpson index	215.66	−104.29	.1179	**.0045**
	Evenness	198.98	−105.70	.1232	**.0037**
Phylogenetic diversity	PD	186.35	−0.02	.2190	**<.001**
	MPD	200.04	−0.16	.2939	**<.001**
	MNTD	213.84	−0.26	.2546	**<.001**
Functional diversity	FRic	148.02	−2.04	.0513	**.0467**
	FEve	147.21	−22.61	.0034	.2781
	FDiv	−32.46	179.96	.0902	**.0119**
	CWM. height	97.50	1.03	.4500	**<.001**
	CWM. LDMC	25.03	303.61	.1105	**.0058**
	CWM. SLA	77.40	0.23	.0345	.0851
	CWM. seed size	111.74	148.22	.0799	**.0171**
	CWM. leaf N	100.78	1.91	.1469	**.0016**
	CWM. leaf P	135.40	0.11	0	.9038

Significant results (*p* < .05) are in bold. PD, Faith's phylogenetic diversity; MPD, mean phylogenetic distance; MNTD, mean nearest taxon phylogenetic distance; FRic, functional richness; FEve, functional evenness; FDiv, functional divergence; CWM, community‐weighted mean; LDMC, leaf dry matter content; SLA, specific leaf area.

**Figure 5:**
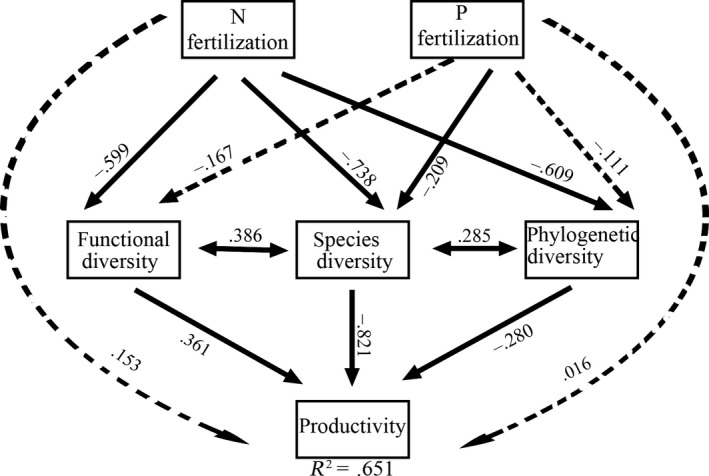
Best fitting structural equation models for using SD, PD, and FD to explaining community biomass in N P fertilization treatment. Thick arrows indicate significant relations; dashed arrows indicate nonsignificant ones. Single‐headed arrows represent causal relationships, while double‐headed arrows represent covarying variables. Numbers on arrows are the standardized path coefficients. Model fit summary: χ^2^ = 0.128, CFI = .991, SRMR = .033

Our SEM successfully elucidated the causal relationships among the variables in our hypothesis model (Appendix S3). The model fits the data well (χ^2^ = 0.128; CFI = .991; TLI = .961; RMSEA = .143; SRMR = .033) and accounted for 65.1% of the variance in community productivity (Figure 5). Our results showed that the effects of N fertilization on SD, PD, and FD were strong, but the effects of P fertilization were weak. N fertilization significantly decreased SD, PD, and FD, while P fertilization only significantly decreased SD but slightly affected PD and FD (Figure 5). We also found that the standardized path coefficients between N or P fertilization and productivity were not significant, which indicated that both N and P had little direct effect on productivity (Figure 5). All three categories of biodiversity significantly influenced community biomass, but the SD was the strongest factor. Finally, the three categories of biodiversity were not independent but covaried with each other (Figure 5, Appendix S4).

## 4 Discussion

### 4.1 Both N and P limit productivity in alpine meadows on the Tibetan Plateau

Many studies have documented that N is the nutrient that most limits productivity in grasslands (Dickson & Gross, 2013; Elser et al., 2007; Humbert, Dwyer, Andrey, & Arlettaz, 2015). Consistent with previous studies (Avolio et al. 2014; Ren et al., 2010), our results also showed that N fertilization increases community productivity in alpine meadow communities. P fertilization alone did not significantly increase productivity, but N + P mixed fertilization had a stronger positive effect on community productivity than N fertilization alone. As Liebig's law of the minimum stated (Verhoeven, Koerselman, & Meuleman, 1996), our results suggested that N is the primary limiting nutrient in alpine meadows, but after N limitation was alleviated by N fertilization, P became the limiting nutrient (Niinemets & Kull, 2005; Van Wijnen & Bakker, 1999).

Consistent with previous studies (Humbert et al., 2015; Li et al., 2015), productivity increased following N fertilization, while species richness, PD, and FRic significantly decreased. However, in our study, N + P mixed fertilization did not result in more biodiversity loss than N fertilization alone, which was in contrast to the results of the classic Park Grass Experiment that considered resource limitation in the context of niche dimensionality and found that multiple nutrient additions led to fewer niche dimensions and decreased diversity (Harpole & Tilman, 2007). A possible explanation might be that a longer time is needed to detect the effect of P fertilization compared to the effect of N fertilization (Niinemets & Kull, 2005).

In our study, CWM for leaf N content increased with N fertilization and CWM for leaf P content increased with P fertilization, which indicated that the growth of herbaceous plants in the alpine meadow was limited by both N and P. In general, leaf N is incorporated into the proteins involved in the photosynthetic machinery, so high leaf N plays an important role in increasing productivity (Wright et al., 2004). In addition, leaf P is found in nucleic acids and lipid membranes, so high leaf P contributes to plant reproduction as opposed to productivity (Wright et al., 2004).

Moreover, fertilization affected the relationships among different soil properties; after N fertilization, the biomass and height of tall, erect grasses greatly increased and overshadowed the understory vegetation and shallow ground, thus reducing the exposure to direct sunlight and decreasing soil temperature. Many studies have emphasized the acidifying effects of using ammonium as a nitrogen fertilizer (Crawley et al., 2005; Yang, Ruijven, & Du, 2011), and consistent with this observation, soil pH had declined from 5.79 ± 0.06 in the control to 5.46 ± 0.11 in the high N fertilization treatment by 2013 in this study.

### 4.2 N fertilization decreased SD, PD, and FD in the alpine meadow community

Many studies have explored the relationships among SD, PD, and FD, and contradictory results have been found (Cadotte et al., 2009; Hevia et al., 2015; Li et al., 2015; Niu et al., 2013). Our results showed that N fertilization decreased SD, PD, and FD, while P fertilization negligibly affected these three biodiversity categories. Intensified light competition may be an important mechanism explaining these results; after N fertilization, biotic interactions may shift from being dominated by belowground competition when soil resources are limited to being dominated by aboveground competition when soil resources are abundant but shading is intense (Hautier, Niklaus, & Hector, 2009; Newman, 1973). Increasing aboveground productivity could intensify aboveground competition for light and decrease light availability in the understory, which could lead to greater mortality or the competitive exclusion of small species (Lamb, Kembel, & Cahill, 2009; Stevens & Carson, 1999). Intensive competition for light was also supported by the finding that the CWM for height increased under N addition (Craine & Dybzinski, 2013; Schellberg & Pontes, 2012). Furthermore, the loss of species richness after N fertilization shortened the total branch length of the cladogram, thus reducing PD in the N‐fertilized community. This congruence makes species richness an appropriate surrogate for PD in alpine meadow communities (Liu et al., 2014).

The mechanism underlying the decrease in FRic after N fertilization was different from that driving the loss of species richness. As all of the species that were used to measure the selected functional traits appeared in all treatments, species loss did not account for the loss of FRic. The main reason for the decrease in FRic was the decline in functional space caused by trait convergence after N fertilization (Schellberg & Pontes, 2012).

### 4.3 Explaining the variation in productivity after fertilization requires the combination of SD, PD, and FD

Two important hypotheses, mass ratio and niche complementarity, have been applied to explain the effect of biodiversity on productivity over the past several decades. According to the mass ratio hypothesis, the most abundant or dominant species are expected to exert the highest impact on productivity (Grime, 1998), so the CWM traits that were obtained by weighing the traits of the species by their relative abundance in a given community (Ackerly, Knight, Weiss, Barton, & Starmer, 2002) are good indicators for testing the mass ratio hypothesis. The overall positive relationships between productivity and CWM traits (CWM height, CWM LDMC, CWM seed size, and CWM leaf N) strongly supported the mass ratio hypothesis (Abul‐Fatih & Bazzaz, 1979; Kröber et al., 2015; Smith & Knapp, 2003). A probable reason is that the abundance of grasses drastically increased following N and N + P fertilization. The increased grasses which have tall height, big seed mass, high LDMC and nitrogen content rapidly grow and produce large biomass after fertilization. On the other hand, FDiv, which captures the degree of divergence in the abundance distribution of species functional traits (Villéger et al., 2008), was closely related to niche differentiation (Mason, Mouillot, Lee, & Wilson, 2005), so the positive relationship between productivity and FDiv could be seen as evidence of niche complementarity (Loreau & Hector, 2001; Tilman, Lehman, et al., 1997). A possible explanation is that fertilization acts as a role of environmental filter, so the species existed in fertilization habitats often have similar characters and occupy similar niches and therefore increase utilization efficiency of limiting resource. Overall, our results suggested that both the mass ratio hypothesis and the niche complementarity hypothesis simultaneously played an important role in explaining the variation in productivity after fertilization but were not mutually exclusive (Hooper et al., 2005; Lohbeck, Poorter, Martínez‐Ramos, & Bongers, 2014b; Loreau, 2000).

However, in contrast to previous studies (Cadotte et al., 2009; Kröber et al., 2015; Lefcheck & Duffy, 2015; Tilman, Knops, et al., 1997), SD explained much more of the variation in productivity after fertilization than FD and PD in this study, which can be explained as follows. First, as mentioned above, the decrease in PD in our study was mainly caused by a loss in species richness, and SD contains the information in PD. Second, as we did not collect data on belowground functional traits, such as root types, rooting depth, or resource requirements, the FD had only limited power to explain the changes in productivity. In future work, data on more functional traits, both aboveground and belowground, should be collected to explore functional diversity.

In our study, the combination of biodiversity indices better explained the variation in productivity than a single biodiversity index. Both the best general linear regression models and the SEM model explained more of the variation in productivity following fertilization than any of the biodiversity indices along, so our results suggest that it is better to use a combination of different aspects of diversity to characterize changes in productivity in an alpine grassland (Lefcheck & Duffy, 2015; Liu et al., 2014). In fact, both FD and PD, which contain information related to the functional traits of species and the phylogenetic relationships in a given community, respectively, are important for elucidating variations in ecosystem functioning (Carboni et al., 2015; Pavoine & Bonsall, 2011), especially productivity. Overall, our study suggests that the combination of FD and PD with SD not only helps explain variations in productivity and reveal the underlying mechanisms (Cadotte, Cardinale, & Oakley, 2008; Liu et al., 2014), but it also plays an important role in predicting the change in the productivity of alpine meadows with increased N deposition.

## 5 Conclusion

A series of field experiments were conducted to explore the limiting nutrients in an alpine meadow and the effects of different biodiversity indices on the variation in productivity following fertilization. Our results showed that N is the primary limiting nutrient alpine meadows, but after N limitation is alleviated by N addition, P becomes the limiting nutrient. We also found that two different hypotheses, mass ratio and niche complementarity, simultaneously explain the changes in our alpine meadow community and were not mutually exclusive. As a result, our findings suggest that combining three aspects of biodiversity is a crucial tool in explaining variations in productivity and understanding the underlying mechanisms.

## Conflict of Interest

None declared.
