# Supplementary material for: Different categories of biodiversity explain productivity variation after fertilization in a Tibetan alpine meadow community
Source: Ecol Evol. 2017 Apr 6;7(10):3464–74. doi: 10.1002/ece3.2723 (PMC5433997; doi:10.1002/ece3.2723)
Supplement: Supplementary file 1 [file ECE3-7-3464-s001.docx]

**Appendix 1** The relative abundance (%) of the 21 common species in different fertilization treatments (FG functional group, G graminoids, F forbs).

| species | FG | treatment | | | | | | | | | |
| --- | --- | --- | --- | --- | --- | --- | --- | --- | --- | --- | --- |
|  |  | CK | N5 | N10 | N15 | P2 | P4 | P8 | N10P2 | N10P4 | N10P8 |
| Agrostis stolonifera | G | 0.37 | 0.39 | 0.22 | 1.24 | 1.07 | 0.77 | 0.86 | 0.00 | 0.15 | 0.31 |
| Allium chysanthum | F | 3.20 | 1.24 | 0.33 | 0.15 | 2.93 | 3.02 | 4.30 | 0.69 | 0.33 | 1.11 |
| Anaphalis hancockii | F | 0.17 | 0.00 | 0.00 | 0.00 | 0.00 | 0.00 | 0.08 | 0.00 | 0.00 | 0.00 |
| Anemone obtusiloba | F | 1.51 | 0.55 | 0.81 | 0.68 | 2.76 | 3.13 | 3.49 | 1.03 | 0.31 | 1.28 |
| Anemone rivularis | F | 6.21 | 7.09 | 13.18 | 6.92 | 6.05 | 4.96 | 6.15 | 8.26 | 5.52 | 3.73 |
| Anemone trullifolia | F | 0.65 | 0.24 | 0.11 | 0.02 | 1.43 | 0.35 | 0.81 | 0.16 | 0.07 | 0.10 |
| Delphinium grandiflorum | F | 0.42 | 0.30 | 0.17 | 0.04 | 0.63 | 0.52 | 0.72 | 0.26 | 0.24 | 0.30 |
| Elymus nutans | G | 8.20 | 31.37 | 32.28 | 50.88 | 6.47 | 9.98 | 7.78 | 23.05 | 38.74 | 38.64 |
| Euphorbia esula | F | 1.04 | 0.20 | 0.19 | 0.14 | 1.31 | 1.61 | 2.18 | 0.31 | 0.52 | 0.98 |
| Gentiana sino-ornata | F | 0.73 | 0.42 | 0.08 | 0.04 | 1.48 | 0.36 | 1.80 | 0.17 | 0.13 | 0.00 |
| Kobresia graminifolia | G | 44.17 | 14.28 | 14.36 | 8.85 | 41.11 | 36.38 | 38.59 | 9.62 | 5.14 | 7.30 |
| Koeleria cristata | G | 0.94 | 2.25 | 0.27 | 0.26 | 0.72 | 0.37 | 0.78 | 0.10 | 0.41 | 0.48 |
| Oxytropis kansuensis | F | 0.29 | 0.01 | 0.00 | 0.00 | 0.93 | 1.10 | 0.38 | 0.07 | 0.00 | 0.05 |
| Poa crymophila | G | 3.03 | 6.70 | 14.31 | 13.27 | 1.76 | 4.22 | 5.43 | 33.38 | 23.87 | 24.98 |
| Poa pratensis | G | 0.21 | 5.36 | 1.86 | 3.93 | 0.62 | 1.75 | 2.26 | 7.62 | 10.94 | 7.65 |
| Potentilla fragarioides | F | 0.61 | 0.05 | 0.18 | 0.04 | 0.63 | 2.49 | 1.34 | 0.08 | 0.02 | 0.04 |
| Ranunculus reptans | F | 0.16 | 0.50 | 0.81 | 0.80 | 0.45 | 0.55 | 0.53 | 1.02 | 0.65 | 1.26 |
| Saussurea neofranchetii | F | 0.01 | 0.00 | 0.40 | 0.43 | 0.27 | 0.32 | 0.10 | 0.00 | 0.00 | 0.00 |
| Saussurea nigrescens | F | 6.11 | 3.10 | 1.42 | 0.84 | 3.09 | 3.86 | 3.06 | 2.50 | 2.16 | 0.79 |
| Saussurea stella | F | 5.82 | 2.04 | 2.37 | 1.64 | 10.71 | 7.34 | 6.98 | 1.36 | 0.85 | 0.70 |
| Veronica didyma | F | 0.18 | 0.27 | 0.12 | 0.06 | 0.26 | 0.78 | 1.04 | 0.06 | 0.14 | 0.33 |

**Appendix 2** Eigenvector scores of species diversity, functional diversity, and phylogenetic diversity on the first and second principal components (PC1 and PC2). Values in parentheses indicate variance accounted for by each axis. The PC1s were used to construct the structural equation models. Explanations of the abbreviations can be found in the description of Table 1.

| species diversity | | | functional diversity | | | phylogenetic diversity | | |
| --- | --- | --- | --- | --- | --- | --- | --- | --- |
|  | PC1(82%) | PC2(17%) |  | PC1(44%) | PC2(16%) |  | PC1(73%) | PC2(19%) |
| Richness | -1.40 | 1.37 | FRic | -1.18 | 0.41 | PD | -1.88 | 0.68 |
| Shannon index | -1.95 | 0.10 | FEve | -0.71 | -0.99 | MPD | -1.93 | 0.47 |
| Simpson index | -1.86 | -0.50 | FDiv | 1.08 | -0.73 | MNTD | -1.59 | -1.38 |
| Evenness | -1.83 | -0.66 | CWM. height | 1.30 | -3.1 |  |  |  |
|  |  |  | CWM. LDMC | 1.36 | -0.24 |  |  |  |
|  |  |  | CWM. SLA | -0.03 | -0.91 |  |  |  |
|  |  |  | CWM. seed size | 1.37 | 0.23 |  |  |  |
|  |  |  | CWM. leaf N | 1.22 | 0.14 |  |  |  |
|  |  |  | CWM. leaf P | -0.12 | -0.98 |  |  |  |

**Appendix 3** The hypothesized structural equation model

We proposed a hypothetical model based on a priori knowledge and then evaluated the model based on empirical data. This model was dependent on the hypothesis that N and P fertilization would directly affect productivity or have indirect effects via different types of diversity (FD, SD, and PD). According to this hypothesis, following N and/or P fertilization, community productivity directly increased due to the alleviation of nutrient limitation (path 1) (Niinemets & Kull 2005; Soudzilovskaia *et al.* 2005; Avolio *et al.* 2014), while N and/or P fertilization also changed species diversity, functional diversity, and phylogenetic diversity (path 2, path 3, and path 4) and thus indirectly affected productivity (path 6, path7, and path 8) (Cadotte *et al.* 2008; Onipchenko *et al.* 2012; Niu *et al.* 2013). For example, following fertilization, short species are often lost due to intensified light competition (path 3) (Li *et al.* 2015a; Yang *et al.* 2015), while the biomass of nutrient-limited grasses drastically increase, which can increase community biomass due to the enhanced availability of limited nutrients (path 7) (Harpole & Tilman 2007; Dickson & Gross 2013; Humbert *et al.* 2015). The responses of PD to fertilization are similar to those of SD because the total length of the cladogram is reduced as species as lost (path 4) (Cadotte *et al.* 2008; Cadotte *et al.* 2009). After fertilization, the biomass of grass species that are phylogenetically similar increases and ultimately determines community biomass (path 8) (Zobel & Pärtel 2008), but the responses of different functional diversity indexes to fertilization are idiosyncratic. Some studies have reported that fertilization increases functional richness and Rao’s index (Niu *et al.* 2013), but several studies have found that fertilization decreases them (path 2). The responses of CWM traits to fertilization depend on the focal functional traits (path 2) (Li *et al.* 2015b). Productivity is affected by FD via two nonexclusive approaches: the mass ratio hypothesis and the niche complementarity hypothesis (path 6) (Butterfield & Suding 2013; Niu *et al.* 2013; Tobner *et al.* 2016). In addition, previous studies have documented that the three categories of biodiversity are not independent but covary with each other (path 5) (Hulot *et al.* 2000; Mayfield *et al.* 2010; Cadotte 2015; Chillo *et al.* 2016).


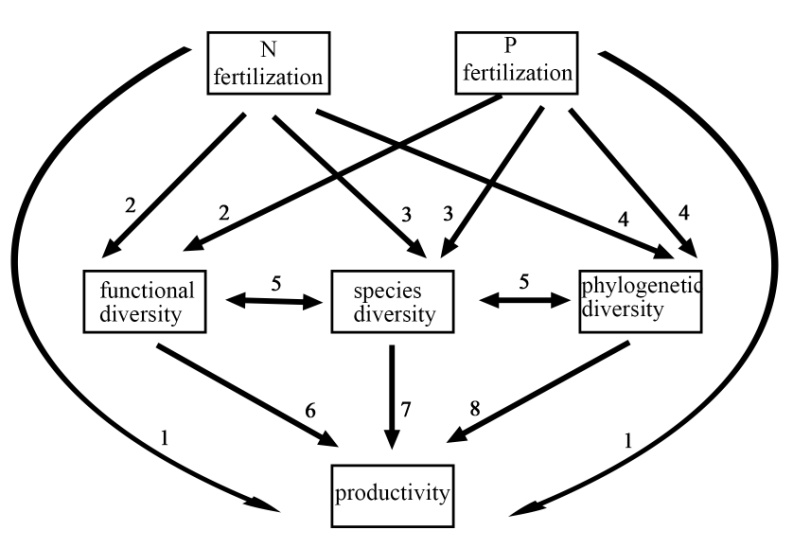


Avolio, M.L., Koerner, S.E., La Pierre, K.J., Wilcox, K.R., Wilson, G.W.T., Smith, M.D. *et al.* (2014). Changes in plant community composition, not diversity, during a decade of nitrogen and phosphorus additions drive above-ground productivity in a tallgrass prairie. *J. Ecol.*, n/a-n/a.

Butterfield, B.J. & Suding, K.N. (2013). Single-trait functional indices outperform multi-trait indices in linking environmental gradients and ecosystem services in a complex landscape. *J. Ecol.*, 101, 9-17.

Cadotte, M.W. (2015). Phylogenetic diversity and productivity: gauging interpretations from experiments that do not manipulate phylogenetic diversity. *Funct. Ecol.*, n/a-n/a.

Cadotte, M.W., Cardinale, B.J. & Oakley, T.H. (2008). Evolutionary history and the effect of biodiversity on plant productivity. *Proceedings of the National Academy of Sciences - PNAS*, 105, 17012-17017.

Cadotte, M.W., Cavender-Bares, J., Tilman, D. & Oakley, T.H. (2009). Using phylogenetic, functional and trait diversity to understand patterns of plant community productivity. *PLoS One*, 4, e5695.

Chillo, V., Ojeda, R.A., Capmourteres, V. & Anand, M. (2016). Functional diversity loss with increasing livestock grazing intensity in drylands: the mechanisms and their consequences depend on the taxa. *J. Appl. Ecol.*, n/a-n/a.

Dickson, T.L. & Gross, K.L. (2013). Plant community responses to long-term fertilization: changes in functional group abundance drive changes in species richness. *Oecologia*, 173, 1513-1520.

Harpole, W.S. & Tilman, D. (2007). Grassland species loss resulting from reduced niche dimension. *Nature*, 446, 791-793.

Hulot, F.D., Lacroix, G., Lescher-Moutoué, F. & Loreau, M. (2000). Functional diversity governs ecosystem response to nutrient enrichment. *Nature*, 405, 340-344.

Humbert, J.-Y., Dwyer, J., Andrey, A. & Arlettaz, R. (2015). Impacts of nitrogen addition on plant biodiversity in mountain grasslands depend on dose, application duration and climate: a systematic review. *Global Change Biol.*, n/a-n/a.

Li, K., Liu, X., Song, L., Gong, Y., Lu, C., Yue, P. *et al.* (2015a). Response of alpine grassland to elevated nitrogen deposition and water supply in China. *Oecologia*, 177, 65-72.

Li, W., Cheng, J.-M., Yu, K.-L., Epstein, H.E., Guo, L., Jing, G.-H. *et al.* (2015b). Plant Functional Diversity Can Be Independent of Species Diversity: Observations Based on the Impact of 4-Yrs of Nitrogen and Phosphorus Additions in an Alpine Meadow. *PLoS ONE*, 10, e0136040.

Mayfield, M.M., Bonser, S.P., Morgan, J.W., Aubin, I., McNamara, S. & Vesk, P.A. (2010). What does species richness tell us about functional trait diversity? Predictions and evidence for responses of species and functional trait diversity to land-use change. *Global Ecol. Biogeogr.*, 19, 423-431.

Niinemets, Ü. & Kull, K. (2005). Co-limitation of plant primary productivity by nitrogen and phosphorus in a species-rich wooded meadow on calcareous soils. *Acta Oecol.*, 28, 345-356.

Niu, K., Choler, P., de Bello, F., Mirotchnick, N., Du, G. & Sun, S. (2013). Fertilization decreases species diversity but increases functional diversity: A three-year experiment in a Tibetan alpine meadow. *Agric., Ecosyst. Environ.*

Onipchenko, V., Makarov, M., Akhmetzhanova, A., Soudzilovskaia, N., Aibazova, F., Elkanova, M. *et al.* (2012). Alpine plant functional group responses to fertiliser addition depend on abiotic regime and community composition. *Plant Soil*, 357, 103-115.

Soudzilovskaia, N., Onipchenko, V., Cornelissen, J. & Aerts, R. (2005). Biomass production, N: P ratio and nutrient limitation in a Caucasian alpine tundra plant community. *Journal of Vegetation Science*, 16, 399-406.

Tobner, C.M., Paquette, A., Gravel, D., Reich, P.B., Williams, L.J. & Messier, C. (2016). Functional identity is the main driver of diversity effects in young tree communities. *Ecol. Lett.*, n/a-n/a.

Yang, Z., Hautier, Y., Borer, E., Zhang, C. & Du, G. (2015). Abundance- and functional-based mechanisms of plant diversity loss with fertilization in the presence and absence of herbivores. *Oecologia*, 1-10.

Zobel, M. & Pärtel, M. (2008). What determines the relationship between plant diversity and habitat productivity? *Global Ecol. Biogeogr.*, 17, 679-684.

**Appendix 4** Correlation matrix between species diversity indices (Richness = Richness; Shannon = Shannon–Wiener index; Simpson = Simpson index; Evenness = Pielou evenness index), phylogenetic diversity (PD = phylogenetic diversity; MPD = mean phylogenetic distance; MNTD = mean nearest taxon phylogenetic distance), and functional diversity (FRic = functional richness; FEve = functional evenness; FDis = functional divergence; CWM = community-weighted mean; LDMC = leaf dry matter content; SLA = specific leaf area). Significant results (p < 0.05) are in bold.

|  | **Richeness** | **Shannon** | **Simpson** | **Evenness** | **PD** | **MPD** | **MNPD** | **FRic** | **FEve** | **FDiv** | **Height** | **LDMC** | **SLA** | **Seed mass** | **Leaf N** |
| --- | --- | --- | --- | --- | --- | --- | --- | --- | --- | --- | --- | --- | --- | --- | --- |
| **Richeness** | **1** |  |  |  |  |  |  |  |  |  |  |  |  |  |  |
| **Shannon** | **0.75** | **1** |  |  |  |  |  |  |  |  |  |  |  |  |  |
| **Simpson** | **0.5** | **0.91** | **1** |  |  |  |  |  |  |  |  |  |  |  |  |
| **Evenness** | **0.43** | **0.92** | **0.94** | **1** |  |  |  |  |  |  |  |  |  |  |  |
| **PD** | **0.71** | **0.85** | **0.68** | **0.74** | **1** |  |  |  |  |  |  |  |  |  |  |
| **MPD** | **0.63** | **0.92** | **0.85** | **0.88** | **0.78** | **1** |  |  |  |  |  |  |  |  |  |
| **MNPD** | **0.58** | **0.45** | 0.24 | **0.27** | **0.47** | **0.53** | **1** |  |  |  |  |  |  |  |  |
| **FRic** | **0.67** | **0.6** | **0.48** | **0.42** | **0.63** | **0.48** | 0.25 | **1** |  |  |  |  |  |  |  |
| **FEve** | 0.2 | 0.21 | 0.18 | 0.18 | 0.22 | 0.17 | 0.17 | 0.19 | **1** |  |  |  |  |  |  |
| **FDiv** | **-0.55** | **-0.7** | **-0.61** | **-0.63** | **-0.7** | **-0.62** | -0.25 | **-0.54** | -0.01 | **1** |  |  |  |  |  |
| **Height** | **-0.89** | **-0.77** | **-0.53** | **-0.53** | **-0.71** | **-0.74** | **-0.67** | **-0.58** | **-0.26** | **0.52** | **1** |  |  |  |  |
| **LDMC** | **-0.59** | **-0.69** | **-0.64** | **-0.61** | **-0.62** | **-0.7** | **-0.36** | **-0.61** | **-0.41** | **0.5** | **0.66** | **1** |  |  |  |
| **SLA** | -0.16 | -0.18 | -0.11 | -0.13 | -0.25 | -0.18 | -0.01 | -0.04 | 0.17 | 0.21 | 0.03 | -0.12 | **1** |  |  |
| **Seed mass** | **-0.54** | **-0.59** | **-0.55** | **-0.5** | **-0.45** | **-0.47** | -0.19 | **-0.47** | **-0.37** | **0.52** | **0.57** | **0.7** | -0.14 | **1** |  |
| **Leaf N** | **-0.55** | **-0.43** | **-0.28** | **-0.26** | **-0.46** | **-0.35** | **-0.29** | **-0.42** | **-0.34** | **0.37** | **0.57** | **0.48** | 0.06 | **0.74** | 1 |

**Appendix 5** The results of generalized linear models. Model selection was based on the comparison of Akaike information criteria (AIC). Significant results (P < 0.05) are in bold. (P: productivity; FRic: functional richness; FDiv: functional divergence; PD: Faith’s phylogenetic diversity; MNTD: mean nearest taxon phylogenetic distance; CWM: community-weighted mean; LDMC: leaf dry matter content (mg/g); SLA: specific leaf area (cm^2^/mg); SM: seed mass (mg))

| **model** | **R^2^** | **P value** | **intercept** | **P value of intercept** | **AIC** |
| --- | --- | --- | --- | --- | --- |
| P~richness+simpson+FRic+FDiv+PD+MNTD +CWM.height+CWM.LDMC+CWM.SM+CWM.leafN | 0.5865 | **<0.001** | 196 | 0.1100 | 336.85 |
| ~richness+simpson+FRic+FDiv+PD+MNTD +CWM.height+CWM.SM+CWM.leafN | 0.5865 | **<0.001** | 195 | 0.0574 | 334.85 |
| ~richness+simpson+FDiv+PD+MNTD+CWM.height+CWM.SM +CWM.leafN | 0.5865 | **<0.001** | 201 | **<0.001** | 332.86 |
| ~richness+simpson+FDiv+PD+CWM.height +CWM.SM+CWM.leafN | 0.5862 | **<0.001** | 196 | **<0.001** | 330.9 |
| ~richness+simpson+FDiv+CWM.height +CWM.SM+CWM.leafN | 0.5856 | **<0.001** | 196 | **<0.001** | 328.99 |
| ~richness+simpson+FDiv+CWM.height  +CWM.SM | 0.5774 | **<0.001** | 191 | **<0.001** | 328.14 |
| ~richness+simpson+FDiv+CWM.height | 0.5721 | **<0.001** | 178 | **<0.001** | 326.88 |
| **~richness+FDiv+CWM.height** | 0.5686 | **<0.001** | 161 | **<0.001** | 325.36 |
